# First seizure in adolescence revealing hidden bilateral porencephalic cysts

**DOI:** 10.1177/2050313X261431037

**Published:** 2026-05-19

**Authors:** Bassem Al Hariri, Mohammed Waleed Ali, Tasneem Abdelrahman, Mohammed H. O. Ali, Wafa Salmah, Farhan Ottakath, Suad Abker, Esra Abdelraouf

**Affiliations:** 1 Department of Medicine, Hamad Medical Corporation, HMGH, Doha, Qatar; 2 College of Medicine, Qatar University, Doha, Qatar; 3 College of Medicine, Weill Cornell Medicine—Qatar, Doha, Qatar; 4 Medical Education Department, Hamad Medical Corporation, Doha, Qatar

**Keywords:** porencephaly, seizure, porencephalic cyst, neuroimaging, adolescence, case report, ILAE classification, antiseizure medications

## Abstract

Porencephaly is a rare neurological disorder characterized by cerebrospinal fluid-filled cavities within the cerebral parenchyma. While typically diagnosed in infancy, silent cases may manifest later in life with new-onset seizures. We report a 14-year-old female with a remote history of encephalitis requiring neurosurgical intervention at 8 months of age who presented with her first generalized tonic–clonic seizure. Laboratory evaluation revealed post-ictal leukocytosis and neutrophilia with otherwise unremarkable serum studies. Neuroimaging demonstrated large bilateral porencephalic cysts communicating with the lateral ventricles, accompanied by surrounding gliosis and mild midline shift. The patient remained seizure-free without immediate antiseizure medication and was initiated on levetiracetam for secondary prophylaxis following multidisciplinary consultation. This case highlights that porencephalic cysts can remain clinically silent for over a decade before presenting first-time seizures in adolescence. A history of early brain insult should prompt thorough neuroimaging evaluation, and adherence to contemporary epilepsy terminology is essential for accurate reporting.

## Introduction

Porencephaly is an uncommon neurological condition characterized by the development of cerebrospinal fluid (CSF)-filled cavities or cysts within the cerebral parenchyma.^1^ These cavities may be congenital, arising from aberrant neuronal migration or prenatal vascular events, or acquired secondary to perinatal or postnatal insults such as ischemia, trauma, hemorrhage, and infection.^2^ The reported incidence is ~3.5/100,000 live births, though the true prevalence in adolescent and adult populations remains uncertain due to limited population-based studies.^3^

Clinical manifestations vary widely, ranging from asymptomatic incidental findings to severe neurological deficits, developmental delay, and epilepsy. Seizures occur in a significant proportion of affected individuals and may be refractory to treatment in ~25% of cases.^4^ While porencephaly is typically diagnosed in infancy or early childhood, delayed presentation with first-time seizures in adolescence or adulthood is rare and may be overlooked without appropriate clinical suspicion and neuroimaging.

We present a case of bilateral porencephalic cysts diagnosed in an adolescent female following a first unprovoked seizure, emphasizing the importance of recognizing remote neurological insults as potential risk factors, adhering to current International League Against Epilepsy (ILAE) terminology, and providing a structured approach to diagnostic evaluation.

## Case presentation

### Patient information and past medical history

A 14-year-old female presented to the emergency department following her first witnessed generalized tonic–clonic seizure. She had been in her usual state of health until the evening of admission when she experienced an abrupt onset of generalized stiffening followed by rhythmic jerking of all extremities, associated with tongue biting and urinary incontinence. The event lasted ~2–3 min, followed by a 15-min post-ictal period of confusion and somnolence. Emergency medical services transported her to the hospital, where she was found to be in a post-ictal state.

Her past medical history was significant for encephalitis requiring neurosurgical intervention at 8 months of age. The specific etiology of the encephalitis and the nature of the neurosurgical procedure could not be ascertained from available records or family recall; however, the family confirmed there were no perioperative complications and the patient achieved complete recovery without residual deficits. She had no prior history of seizures, developmental delays, or focal neurological deficits. There was no family history of epilepsy or neurodevelopmental disorders.

### Clinical findings and neurological examination

On arrival, vital signs were normal: afebrile (36.6 °C), blood pressure 108/76 mmHg, heart rate 76 beats/min, respiratory rate 18 breaths/min, and oxygen saturation 98% on room air. A second generalized tonic–clonic seizure was witnessed by the treating team in the emergency department and terminated spontaneously within 90 s.

Neurological examination performed 30 min post-ictal revealed

- *Glasgow Coma Scale:* 15/15.
- *Cranial nerves:* Intact II–XII, normal pupillary reflexes, no nystagmus, facial symmetry, intact gag reflex.
- *Motor system:* Normal bulk and tone, power 5/5 in all four limbs, no pronator drift.
- *Sensory system:* Intact light touch, pinprick, proprioception, and vibration.
- *Reflexes:* Symmetrical 2+ deep tendon reflexes, flexor plantar responses bilaterally.
- *Coordination:* Normal finger-to-nose and heel-to-shin testing.
- *Gait:* Not formally assessed due to post-ictal state, but patient able to move all extremities purposefully.

Cardiopulmonary, abdominal, and skin examinations were unremarkable.

### Diagnostic assessment

#### Laboratory investigations

Initial laboratory studies (obtained 35 min post-ictal) revealed post-ictal leukocytosis (white blood cell count 15.2 × 10^3^/µL) with neutrophilia (absolute neutrophil count 12.8 × 10^3^/µL). Serum bicarbonate was marginally low at 20 mmol/L, and glucose was mildly elevated at 5.7 mmol/L, consistent with a physiological stress response. All other parameters including renal function, electrolytes, liver enzymes, coagulation profile, C-reactive protein (2 mg/L), and adjusted calcium were within normal limits. Serial laboratory monitoring demonstrated normalization of leukocytosis and bicarbonate by hospital day 2 (Table 1).

**Table 1.** Laboratory results.

Parameter	On discharge day, September 15, 2025 (04:20)	Second day of admission, September 14, 2025 (05:17)	First day of admission, September 13, 2025 (19:52)	Normal range
WBCs (×10^6^/µL)	7.0	12.5	15.2	4.0–10.0
RBCs (×10^6^/µL)	4.6	4.5	4.6	3.8–4.8
Hgb (g/dL)	11.8	11.8	12.3	12.0–15.0
Hct (%)	37.0	36.0	36.5	36.0–46.0
MCV (fL)	81.1	79.5	78.7	83.0–101.0
MCH (pg)	25.9	26.0	26.5	27.0–32.0
MCHC (g/dL)	31.9	32.8	33.7	31.5–34.5
RDW-CV (%)	13.3	13.2	13.0	11.6–14.0
Platelet (×10^3^/µL)	279	293	305	150–410
MPV (fL)	10.4	10.3	10.3	9.6–12.0
ANC Auto ()	3.3	9.6	12.8	2.0–7.0
Lymphocyte auto (#)	3.2	2.2	1.6	1.0–3.0
Monocyte auto (#)	0.4	0.6	0.7	0.2–1.0
Eosinophil auto (#)	L 0.09	L 0.02	L0.04	0.02–0.50
Basophil auto (#)	L 0.02	L0.01	L0.02	0.02–0.10
Neutrophil auto (%)	47.6	76.5	84.6	
Lymphocyte auto (%)	45.2	18.0	10.4	
Monocyte auto (%)	5.6	5.2	4.6	
Eosinophil auto (%)	1.3	0.2	0.3	
Basophil auto (%)	0.3	0.1	0.1	
Coagulation				
Prothrombin time			12.1	9.4–12.5
INR			1.1	>4.9
APTT			24.7	25.1–36.5
Blood chemistry				
Urea (mmol/L)	3.6	3.4	3.8	2.5–7.8
Creatinine (µmol/L)	53	48	71	44–80
Sodium (mmol/L)	138	134	137	133–146
Potassium (mmol/L)	3.7	4.1	3.7	3.5–5.3
Chloride (mmol/L)	106	104	104	95–108
Bicarbonate (mmol/L)	22	19	20	22–29
Calcium			2.32	
Adjusted calcium			2.36	2.20–2.60
Bilirubin (T)		5	3	0–21
Total protein (g/L)		70	77	60–80
Albumin (g/L)		35	38	35–50
Alk phos (U/L)		85	89	35–104
ALT (U/L)		13	13	0–33
AST (U/L)		17	20	0–32
Glucose			5.7	
CRP		2.2	2	0.0–5.0
PrPH Ven–POC			7.380	
PCO2ven–PCO			37	

ANC: absolute neutrophile count.

#### Neuroimaging

**Figure 1.**
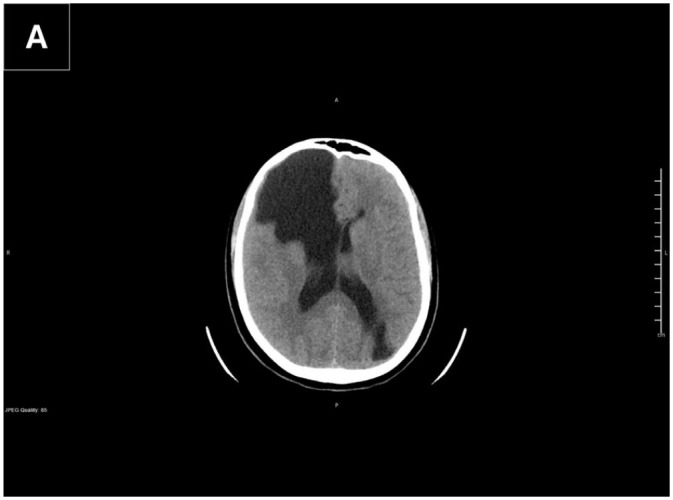
(a) Axial non-contrast CT of the head demonstrates a large porencephalic cyst in the right frontal lobe communicating with the right lateral ventricle, associated with ex vacuo ventricular dilatation and chronic calvarial remodeling. A second porencephalic cyst is seen in the left occipital lobe communicating with the occipital horn of the left lateral ventricle. No acute intracranial hemorrhage or significant mass effect is identified. R/L orientation markers are visible. CT: computed tomography.

Non-contrast computed tomography of the head (Figure 1(a)) revealed two large CSF-attenuation cysts. The larger cyst occupied the right frontal lobe, communicating with the frontal horn of the right lateral ventricle with associated ex vacuo dilatation. A second cyst was identified in the left occipital lobe, communicating with the occipital horn of the left lateral ventricle.

**Figure 2.**
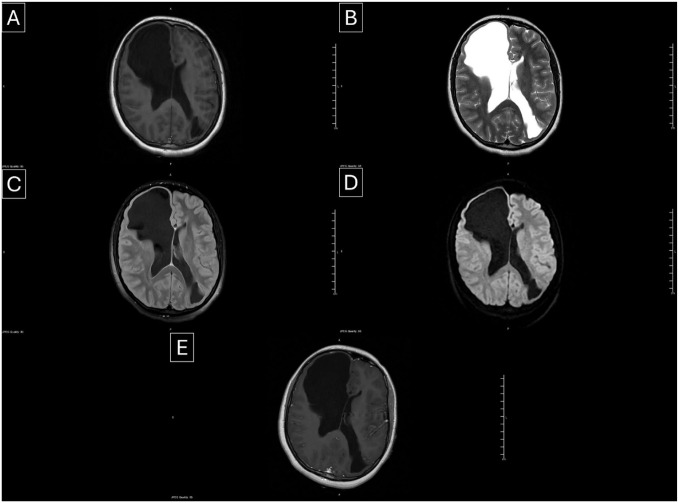
(a–e) Axial MRI of the brain demonstrates bilateral porencephalic cysts: (a, T1-weighted) CSF-intense cystic lesion in the right frontal lobe communicating with the frontal horn of the right lateral ventricle, with associated cortical thinning. A similar CSF-intense cyst is seen in the left occipital lobe communicating with the occipital and temporal horns of the left lateral ventricle, (b, T2-weighted) the cysts appear hyperintense, clearly delineating their extent and ventricular communication, (c, FLAIR) perilesional hyperintensity consistent with gliosis is seen adjacent to the right frontal cyst, while no surrounding gliosis is noted around the left occipital cyst, (d, diffusion-weighted imaging) no diffusion restriction is identified within either lesion, and (e, post-contrast T1-weighted) no abnormal enhancement is observed. Mild mass effect with subtle leftward midline shift is noted. R/L orientation markers are visible in all panels. CSF: cerebrospinal fluid; MRI: magnetic resonance imaging.

Brain magnetic resonance imaging (MRI) with and without contrast (Figure 1(b)–(e)) confirmed and characterized these findings in greater detail (Figure 2):

- *Right frontal cyst:* Measured 13.0 × 6.5 × 5.6 cm (AP × CC × transverse), demonstrating CSF signal intensity on all sequences, no diffusion restriction, and no pathological enhancement. Broad communication with the right lateral ventricle was observed, with adjacent T2/FLAIR hyperintensity consistent with chronic gliosis. Mild mass effect with 3–4 mm leftward midline shift was noted.
- *Left occipital cyst:* Measured 5.5 × 3.4 × 3.2 cm, with similar imaging characteristics, communicating with the occipital and temporal horns of the left lateral ventricle. No surrounding gliosis was identified.
- *Additional findings:* Both cysts demonstrated thinning of the overlying cortex and mild calvarial remodeling, indicating chronicity. There was no hydrocephalus, hemorrhage, or contrast enhancement to suggest active inflammation or neoplasm.

#### Electroencephalography

**Figure 3.**
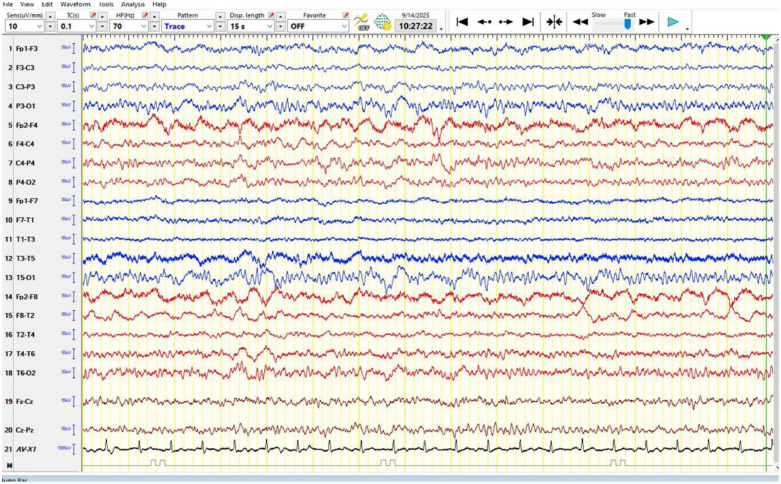
Routine awake EEG (International 10–20 system) demonstrates a symmetric 9 Hz posterior dominant alpha rhythm reactive to eye opening. Occasional irregular delta slowing is noted over the right frontocentral region, with more frequent irregular delta activity over the left posterior region. No epileptiform discharges or electrographic seizures are observed. Findings are consistent with focal cerebral dysfunction corresponding to the regions of structural abnormality. EEG: electroencephalography.

A routine awake electroencephalography (EEG) performed on hospital day 2 using the International 10–20 system demonstrated a symmetric 9 Hz posterior dominant alpha rhythm that reacted appropriately to eye opening. Occasional irregular delta slowing was noted over the right frontocentral region, with more frequent irregular delta activity over the left posterior region. No epileptiform discharges, spike-wave activity, or electrographic seizures were observed. These findings are consistent with focal cerebral dysfunction corresponding to the regions of structural abnormality identified on neuroimaging and do not indicate an active epileptic focus (Figure 3).

#### Temporal context and rationale for imaging

Neuroimaging was obtained emergently following the second witnessed seizure to exclude acute intracranial pathology (hemorrhage, mass, infection) in a patient with no prior seizure history. The finding of bilateral porencephalic cysts was incidental to this evaluation and prompted further characterization with MRI.

#### Timeline of clinical events

To provide a clear chronological overview, key events from infancy through follow-up are summarized in Table 2.

**Table 2.** Clinical timeline.

Date/age	Event
8 months of age	Encephalitis; neurosurgical intervention (details unknown)
8 months–14 years	Asymptomatic period; no seizures, normal development
September 13, 2025 (19:52)	First generalized tonic–clonic seizure at home; EMS transport
September 13, 2025 (evening)	Second witnessed seizure in ED; initial laboratory studies obtained
September 14, 2025	Admission; EEG performed; MRI brain obtained
September 15, 2025 (04:20)	Discharged on levetiracetam 500 mg twice daily
4-week follow-up	Seizure-free; therapeutic levetiracetam level; no adverse effects; adherence confirmed
6-week follow-up	Follow-up EEG: interval resolution of focal slowing
6-month follow-up (planned)	Repeat neuroimaging surveillance

EEG: electroencephalography; MRI: magnetic resonance imaging.

### Therapeutic intervention and hospital course

The patient was admitted for continuous cardiorespiratory and neurological monitoring. She remained seizure-free without initiation of antiseizure medication (ASM) for 48 h post admission. Following multidisciplinary consultation with neurology and neurosurgery, the decision was made to initiate secondary prophylaxis given the presence of large bilateral structural lesions and the occurrence of two unprovoked generalized seizures.

Levetiracetam 500 mg twice daily was selected due to its favorable side effect profile, minimal drug interactions, rapid titration, and established efficacy in focal and generalized onset seizures associated with structural brain abnormalities. Alternative agents (e.g. valproate, carbamazepine) were considered but deferred due to potential adverse effects and the patient’s childbearing potential.

Neurosurgical evaluation concluded that there were no indications for surgical intervention at presentation, as the cysts were chronic, demonstrated no mass effect requiring fenestration, and were not associated with refractory seizures or progressive neurological deficit. The patient and family were counseled regarding the benign natural history of asymptomatic porencephalic cysts and the rationale for medical management.

### Follow-up and outcomes

The patient was discharged on hospital day 3, neurologically intact and hemodynamically stable. At 4-week follow-up, she remained seizure-free and reported no adverse effects from levetiracetam. Adherence was confirmed, and serum levetiracetam level was within therapeutic range. A follow-up EEG at 6 weeks demonstrated interval resolution of focal slowing. Ongoing care includes pediatric neurology and neurosurgery surveillance, with repeat imaging planned at 6 months or sooner if clinically indicated.

## Discussion

Porencephalic cysts are rare CSF-filled cavities within the brain parenchyma that may result from genetic (porencephaly gene mutations, COL4A1/A2) or encephaloclastic (vascular, infectious, traumatic) mechanisms.^1,2^ While typically diagnosed in infancy, our case demonstrates that clinically silent cysts can remain undetected for over a decade before manifesting with new-onset seizures in adolescence.

### Comparison with existing literature

Most reported porencephaly cases are unilateral and diagnosed in early childhood.^4^ Bilateral involvement, as seen in our patients, is distinctly uncommon. A 2021 case by Dominguez et al. described a 13-year-old female who developed a porencephalic cyst 2.5 months following endoscopic third ventriculostomy.^5^ In contrast, our patient remained asymptomatic for 13 years after her infantile neurosurgical intervention, representing one of the longest reported latency periods. Qureshi et al. reported a 56-year-old male with a first-time seizure secondary to a porencephalic cyst, with a history of infantile intracerebral hemorrhage.^6^ Our case parallels this delayed presentation but is unique due to the patient’s adolescent age, bilateral cyst location, and remote history of encephalitis rather than hemorrhage.

### Etiology and pathogenesis

Although the precise etiology in our patient remains uncertain due to unavailable infantile records, the combination of encephalitis and neurosurgical intervention in infancy provides a plausible encephaloclastic basis. The absence epilepsy, lack of COL4A1-associated systemic features (cataracts, renal involvement, hemorrhagic stroke), and chronic imaging findings (calvarial remodeling, gliosis) favor an acquired, remote insult rather than an active genetic or progressive process.

### Diagnostic considerations and ILAE terminology

This case underscores the importance of accurate seizure classification. Using ILAE 2017 terminology, the patient’s semiology is best described as bilateral tonic–clonic seizure (previously “generalized tonic–clonic”) of focal onset (given the structural lesions and focal EEG slowing).^7^ We have revised our manuscript to replace all instances of “antiepileptic drugs” with “ASMs,” reflecting the contemporary understanding that these agents suppress seizures without modifying the underlying disease course.^8^

### Management rationale

There are no randomized controlled trials guiding ASM prophylaxis in porencephaly. Our decision to initiate levetiracetam was based on expert consensus for secondary prevention following two unprovoked seizures in the setting of a structural brain lesion.^9^ Levetiracetam was favored over enzyme-inducing ASMs due to its lack of hepatic metabolism, minimal drug interactions, and favorable cognitive profile particularly important in an adolescent population. Surgical intervention (cyst fenestration, shunting) is reserved for patients with refractory seizures, progressive hydrocephalus, or significant mass effect;^10^ none of these indications were present in our case.

### Limitations

We acknowledge several limitations. First, the precise etiology and surgical details of the patient’s infantile encephalitis could not be verified, representing an unavoidable information gap. Second, genetic testing for COL4A1/A2 mutations was not performed; while the clinical and imaging phenotype is consistent with acquired porencephaly, inherited forms cannot be definitively excluded. Third, follow-up duration is currently short; long-term data regarding seizure freedom and cyst stability are pending.

### Educational value and reporting adherence

This case reinforces that a remote history of CNS infection or surgery—even in the distant past—should not be dismissed in patients presenting with new-onset seizures. We have structured this report in accordance with the Case Report (CARE) guidelines to ensure completeness and transparency (Supplemental Material).^11^

## Conclusion

Porencephalic cysts are rare neurological lesions that can remain clinically silent for years before presenting new-onset seizures in adolescence. This case highlights the importance of maintaining a high index of suspicion in patients with a history of remote CNS insults, utilizing MRI as the imaging modality of choice, and adhering to current ILAE terminology and epilepsy classification. Multidisciplinary management including neurology, neurosurgery, and primary care is essential to optimizing outcomes. Clinicians should be aware that bilateral cysts can present with first-time seizures after prolonged latency, and that conservative medical management with ASMs is often appropriate in the absence of refractory epilepsy or progressive mass effect.

## Supplemental Material

sj-docx-1-sco-10.1177_2050313X261431037 – Supplemental material for First seizure in adolescence revealing hidden bilateral porencephalic cysts Supplemental material, sj-docx-1-sco-10.1177_2050313X261431037 for First seizure in adolescence revealing hidden bilateral porencephalic cysts by Bassem Al Hariri, Mohammed Waleed Ali, Tasneem Abdelrahman, Mohammed H. O. Ali, Wafa Salmah, Farhan Ottakath, Suad Abker and Esra Abdelraouf in SAGE Open Medical Case Reports
