# Supplementary material for: First seizure in adolescence revealing hidden bilateral porencephalic cysts
Source: SAGE Open Med Case Rep. 2026 May 19;14:2050313X261431037. doi: 10.1177/2050313X261431037 (PMC13187386; doi:10.1177/2050313X261431037)
Supplement: sj-docx-1-sco-10.1177_2050313X261431037 – Supplemental material for First seizure in adolescence revealing hidden bilateral porencephalic cysts [file sj-docx-1-sco-10.1177_2050313X261431037.docx]

| **Topic** | **Item** | **Checklist Item Description** | **Line / Page Reference** |
| --- | --- | --- | --- |
| **Title** | 1 | The words "case report" should be in the title along with the area of focus | Title Page |
| **Key Words** | 2 | Four to seven key words—include "case report" as one of the key words | Abstract (Keywords) |
| **Abstract** | 3a | Background: What does this case report add to the medical literature? | Abstract (paragraph 1) |
|  | 3b | Case summary: chief complaint, diagnoses, interventions, and outcomes | Abstract (paragraph 2) |
|  | 3c | Conclusion: What is the main "take-away" lesson from this case? | Abstract (final sentence) |
| **Introduction** | 4 | The current standard of care and contributions of this case—with references (1-2 paragraphs) | Introduction (entire) |
| **Timeline** | 5 | Information from this case report organized into a timeline (table or figure) | **Table 2** (Case Presentation) |
| **Patient Information** | 6a | De-identified demographic and other patient or client specific information | Case Presentation (Patient Information) |
|  | 6b | Chief complaint—what prompted this visit? | Case Presentation (first sentence) |
|  | 6c | Relevant history including past interventions and outcomes | Case Presentation (Past Medical History) |
| **Physical Exam** | 7 | Relevant physical examination findings | Case Presentation (Neurological Examination) |
| **Diagnostic Assessment** | 8a | Evaluations such as surveys, laboratory testing, imaging, etc. | Case Presentation (Diagnostic Assessment) |
|  | 8b | Diagnostic reasoning including other diagnoses considered and challenges | Case Presentation (Temporal Context); Discussion (Etiology) |
|  | 8c | Consider tables or figures linking assessment, diagnoses and interventions | Table 1, Figure 1 |
|  | 8d | Prognostic characteristics where applicable | Discussion (Management Rationale, Limitations) |
| **Interventions** | 9a | Types such as life-style recommendations, treatments, medications, surgery | Case Presentation (Therapeutic Intervention) |
|  | 9b | Intervention administration such as dosage, frequency and duration | Levetiracetam 500 mg BID (Therapeutic Intervention) |
|  | 9c | Note changes in intervention with explanation | No changes (N/A) |
|  | 9d | Other concurrent interventions | None (N/A) |
| **Follow-up and Outcomes** | 10a | Clinician assessment (and patient or client assessed outcomes when appropriate) | Case Presentation (Follow-up and Outcomes) |
|  | 10b | Important follow-up diagnostic evaluations | Follow-up EEG (same section) |
|  | 10c | Assessment of intervention adherence and tolerability, including adverse events | Adherence confirmed, no adverse effects (same section) |
| **Discussion** | 11a | Strengths and limitations in your approach to this case | Discussion (Limitations) |
|  | 11b | Specify how this case report informs practice or Clinical Practice Guidelines (CPG) | Discussion (Educational Value) |
|  | 11c | How does this case report suggest a testable hypothesis? | Discussion (Educational Value, final sentence) |
|  | 11d | Conclusions and rationale | Conclusion (entire) |
| **Patient Perspective** | 12 | When appropriate include the assessment of the patient or client on this episode of care | **Not obtained / Not applicable** |
| **Informed Consent** | 13 | Informed consent from the person who is the subject of this case report is required by most journals | Declarations (Consent for Publication) |
| **Additional Information** | 14 | Acknowledgement section; Competing Interests; IRB approval when required | Declarations (all) |
